# Genetic Ancestry, Self-Reported Race and Ethnicity in African Americans and European Americans in the PCaP Cohort

**DOI:** 10.1371/journal.pone.0030950

**Published:** 2012-03-27

**Authors:** Lara E. Sucheston, Jeannette T. Bensen, Zongli Xu, Prashant K. Singh, Leah Preus, James L. Mohler, L. Joseph Su, Elizabeth T. H. Fontham, Bernardo Ruiz, Gary J. Smith, Jack A. Taylor

**Affiliations:** 1 Department of Cancer Prevention and Control, Roswell Park Cancer Institute, Buffalo, New York, United States of America; 2 Department of Epidemiology, University of North Carolina, Chapel Hill, North Carolina, United States of America; 3 Lineberger Comprehensive Cancer Center, University of North Carolina, Chapel Hill, North Carolina, United States of America; 4 Epidemiology Branch and Laboratory of Molecular Carcinogenesis, National Institute of Environmental Health Sciences, Research Triangle Park, North Carolina, United States of America; 5 Department of Genetics and Pharmacology, Roswell Park Cancer Institute, Buffalo, New York, United States of America; 6 Department of Urology, Roswell Park Cancer Institute, Buffalo, New York, United States of America; 7 Department of Urology, State University of New York at Buffalo, Buffalo, New York, United States of America; 8 Division of Cancer Control and Population Sciences, National Cancer Institute, Bethesda, Maryland, United States of America; 9 Department of Epidemiology, School of Public Health, Louisiana State University Health Sciences Center, New Orleans, Louisiana, United States of America; 10 Department of Pathology, Louisiana State University Health Sciences Center, New Orleans, Louisiana, United States of America; University of Utah, United States of America

## Abstract

**Background:**

Family history and African-American race are important risk factors for both prostate cancer (CaP) incidence and aggressiveness. When studying complex diseases such as CaP that have a heritable component, chances of finding true disease susceptibility alleles can be increased by accounting for genetic ancestry within the population investigated. Race, ethnicity and ancestry were studied in a geographically diverse cohort of men with newly diagnosed CaP.

**Methods:**

Individual ancestry (IA) was estimated in the population-based North Carolina and Louisiana Prostate Cancer Project (PCaP), a cohort of 2,106 incident CaP cases (2063 with complete ethnicity information) comprising roughly equal numbers of research subjects reporting as Black/African American (AA) or European American/Caucasian/Caucasian American/White (EA) from North Carolina or Louisiana. Mean genome wide individual ancestry estimates of percent African, European and Asian were obtained and tested for differences by state and ethnicity (Cajun and/or Creole and Hispanic/Latino) using multivariate analysis of variance models. Principal components (PC) were compared to assess differences in genetic composition by self-reported race and ethnicity between and within states.

**Results:**

Mean individual ancestries differed by state for self-reporting AA (*p* = 0.03) and EA (*p* = 0.001). This geographic difference attenuated for AAs who answered “no” to all ethnicity membership questions (non-ethnic research subjects; *p* = 0.78) but not EA research subjects, *p* = 0.002. Mean ancestry estimates of self-identified AA Louisiana research subjects for each ethnic group; Cajun only, Creole only and both Cajun and Creole differed significantly from self-identified non-ethnic AA Louisiana research subjects. These ethnicity differences were not seen in those who self-identified as EA.

**Conclusions:**

Mean IA differed by race between states, elucidating a potential contributing factor to these differences in AA research participants: self-reported ethnicity. Accurately accounting for genetic admixture in this cohort is essential for future analyses of the genetic and environmental contributions to CaP.

## Introduction

Prostate cancer (CaP) is the most common cancer diagnosed in men and the second leading cause of cancer death among men in the US, with African American (AA) men having substantially higher CaP incidence and mortality rates than men self-reporting as European American (EA). CaP is a multifactorial disease with both genetic and environmental components. Familial aggregation has been demonstrated in both AA and EA [1], [2]. A positive family history is one of the strongest known risk factors for CaP and quantitative estimates from twin studies indicate that 42% of CaP cases may have a heritable component [3], which is stronger than any other common cancer [4], [5], [6], [7]. CaP linkage and association studies have identified many genetic variants associated with CaP, although EA and AA may not share all loci as risk factors [8], [9], [10], [11], and replication of these findings has sometimes been inconsistent [12]. Thus, while genome-wide association studies provide a powerful tool for investigating possible genetic factors that may contribute to the health disparities observed among different racial and ethnic populations, association studies can more easily identify disease-associated alleles when study groups are genetically similar, i.e. share a similar ancestral background [1]. In fact, failure to adjust for genetic race and ethnicity in analyses of genetic susceptibility to disease incidence and aggressiveness has been shown to reduce power and increase false positive findings [13], [14], [15]. However, defining genetically similar groups can be challenging in clinical and epidemiologic studies and there is not, at present, a single accepted method used to characterize race and/or ethnicity [3], [4], [5], [6].

Two main methods have been used to summarize individual ancestry in population-based studies: (a) self-identified race and ethnicity and (b) Ancestry Informative Markers (AIMs) genotyped in the population under study [7], [8], [16]. Two measures of genetic ancestry can be derived from AIMs: individual ancestry (IA) percentages, which indicate how much of a person's genome is from a particular ancestral group, and a series of principal components (PC), which quantify an individual's genetic composition. While IA percentages and PC are closely related, self-reported race and AIMs derived measurements (IA and PC) provide different information about an individual, as evidenced by the fact that self-identified racial categories do not consistently predict genetic ancestry (and vice versa). The difference between genetic ancestry and self-reported race could be due to the ability of genetic markers to describe and distinguish populations and the ancestry of the populations under consideration [16], [17], [18], [19], [20]. However, it is important to remember that self-reported race and IA/PCs are derived from genetic information (AIMs) that provides more precise estimates of an individual's ancestral continent(s) of origin, while self-reported race provides (additional) information on social, dietary, and environmental exposures that may be relevant to disease risk. In addition, self-reported race and ethnicity may vary over time or depend upon the context in which questions on race are asked. [12], [21]. Regardless of the source of the difference between these two measures, one does not perfectly predict the other and this relationship is study specific [16], [17], [22], [23].

The PCaP cohort is racially and ethnically diverse and was designed to investigate the contribution that social, biological, and environmental factors make to observed racial differences in CaP mortality in AAs and EAs in the United States. Specifically AAs from North Carolina and Louisiana have, respectively, some of the highest and lowest AA CaP mortality rates in the United States, while EA men in the two states have similar CaP mortality that is less than either AA group. One PCaP research hypothesis was that the higher CaP mortality rates could, in part, reflect a higher proportion of African ancestry in AAs from North Carolina vs AAs in Louisiana. However, the genetic background of PCaP research subjects must be carefully characterized in order to examine the molecular and genetic factors associated with susceptibility to aggressive CaP and other CaP phenotypes. The proportion of African, European and Asian genetic ancestry was measured to evaluate differences in African ancestry across and within regions by ethnicity using individual ancestry estimates [7], [8]. We hypothesized that the proportions of African ancestry in self-reporting AA research subjects would differ by state due to admixture events with French populations experienced in Louisiana but not North Carolina [24], [25]. We anticipated that these differences would be highlighted within Louisiana, such that self-reported AA research participants claiming membership to Cajun and/or Cajun/Creole populations would have significantly different mean individual ancestry estimates from AA research participants that did not belong to an ethnic group [24], [25].

## Methods

### Research Subjects

Written informed consent was obtained from all research subjects prior to blood and questionnaire collection. The study was approved by the University of North Carolina at Chapel Hill (UNC-CH) and Louisiana State University Health Sciences Center (LSUHSC) Institutional Review Boards and the Department of Defense Human Subjects Research Review Board. PCaP is a multidisciplinary study of racial/ethnic differences in social, host, and tumor-specific factors on CaP aggressiveness and outcome [26]. The population-based sample of incident CaP cases is composed of 2106 men (1043 AA and 1063 EA) with genetic data, with 1176 research subjects from 42 counties in central and eastern North Carolina and 930 research subjects from 21 parishes in Louisiana (13 parishes surrounding New Orleans and 8 parishes in southern Louisiana, which were added as a result of population displacement due Hurricane Katrina which occurred on August 29, 2005). Study nurses administered structured questionnaires and collected blood and other biospecimens during an in-home visit. Self-reported ethnicity was collected prior to race information so that the ethnic groups were defined independent of race. The following series of questions were used: “Do you consider yourself to be Hispanic or Latino?”, “Do you consider yourself to be Cajun?” with a sub question, “Was French spoken in your home when you were a child?”, and “Do you consider yourself to be Creole?” Yes, no, don't know, refused were available responses to questions on ethnicity. Self-identified race was established using the following open-ended question: “What is your race?” Men considering themselves to be either African American/Black or European American/Caucasian American/Caucasian/White were eligible for the study. Complete race information was available on all research subjects. One individual was missing ethnicity information for all questions and 1 individual was missing information on questions regarding both Hispanic and Creole ethnicity membership; 5, 11 and 23 individuals either did not respond or responded “don't know” to the questions regarding Hispanic/Latino, Cajun, and Creole ethnicity, respectively. Of the 2106 research participants with available genetic and race data, 2065 men (1022 AA and 1043 EA) responded to all ethnicity questions.

### Ancestry Informative Markers (AIMs)

IA estimates obtained using as few as 30 AIMs shows a correlation of approximately .9 with true individual ancestry estimated using a much larger genome wide panel [20], [27]. Fifty AIMs were selected using allele frequency information from HapMap phase I+II genotype data (http://hapmap.ncbi.nlm.nih.gov) from three populations: Yoruba individuals in Ibadan, Nigeria (YRI) represented African ancestry, Utah residents with Northern and Western European ancestry collected by the Centre d'Etude du Polymorphisme Humain (CEU), represented European ancestry and Japanese individuals from Tokyo, Japan (JPT) and Han, China (CHB), the latter two groups collectively represented Asian (ASI) ancestry. SNPs were selected as follows: twenty-five SNPs had a variant allele frequency (VAF) = 0 in CEU, were rare in ASI, VAF<0.01, but common in YRI with VAF>0.65 and AA VAF>0.25. The other half of the selected SNPs had a VAF = 0 in YRI, were rare in ASI (VAF<0.05), but common in CEU (VAF>0.5). Selected SNPs were at least 10 million base pairs apart.

### Genotyping

DNA was extracted from fresh peripheral blood mononuclear cells (PBMCs), or immortalized lymphoblasts. Genotyping was performed on an Illumina platform at the Center for Inherited Disease Research (CIDR) at Johns Hopkins University as part of a larger genotyping effort [24]. Data quality was monitored by the inclusion of 22 blind duplicates, and 8 CEU and 11 YRI trios from Hapmap (http://hapmap.ncbi.nlm.nih.gov). Forty SNPs passed quality control and greater than 98.6% genotyping success was achieved for all research subjects.

### Individual Ancestry Estimation

Allele frequencies were estimated using maximum likelihood methods. IA proportions for self-reporting AA and EA research subjects were estimated using a Bayesian Markov Chain Monte Carlo (MCMC) clustering algorithm implemented in STRUCTURE 2.3.1 [28], [29]. Publicly available genotypes were included from YRI, CEU and ASI ancestral populations in the STRUCTURE procedure. STRUCTURE was run multiple times under the admixture and independent allele frequency model (constant λ = 1.0) using 100,000 burn-ins and 100,000 iterations after burn-in assuming K = 1,2 and 3 populations. Likelihood tests were performed to determine the appropriate number of populations.

### Comparison of mean Individual Ancestry estimates between and within geographic regions

R Statistical software was used for all analyses comparing research subjects between and within North Carolina and Louisiana (http://cran.r-project.org). Tests of mean CEU and YRI ancestry estimate differences between states were performed using one-way multivariate analysis of variance (MANOVA). As follow-up to the multivariate model, Welch's t-tests were used to test the null hypothesis that there was no difference in mean YRI estimates by state. Within race MANOVA models were constructed to compare ancestry estimates of individuals reporting no ethnicity with those of each ethnic group (Cajun, Creole and Hispanic/Latino). All ethnic analyses were limited to research subjects from Louisiana because only 2% of research subjects reporting ethnicity membership were from North Carolina.

### Principal Component analyses

Principal components analyses were performed as described in Price et al., 2006 [29]. Principal components (PC) for each race were compared by geographic location and within race across ethnicities graphically and using Wilcoxon rank sum tests.

## Results

Table S1 contains SNPs and their corresponding allele frequencies in AA and EA research subjects.

### Individual ancestry estimates

The multiple STRUCTURE runs yielded likelihood and IA estimates that were very close in value. Likelihood estimates consistently favored a model with two populations (CEU and YRI); these two populations were used in all subsequent statistical calculations. Table 1 contains mean percentage IA by self-reported race and location for all three reference groups and mean IA estimates for individuals responding “no” to all ethnicity questions (non-ethnic EA and non-ethnic AA). Mean CEU and YRI (YRI only) ancestry in participants self-reporting as Black/African American did vary significantly between North Carolina and Louisiana, *p*<0.03 (*p*<0.007). However, there was no significant mean difference in IA by state for either CEU or YRI (YRI only) in non-ethnic AA research subjects, *p* = 0.87 (p = 0.78). CEU and YRI ancestry estimates differed by state, in both all and non-ethnic self-reporting EA research subjects, *p* = 0.001 and *p* = 0.002, respectively. When comparing only the mean proportion of YRI between states in all men self-reporting as EA, there were also significant differences (*p*<0.0006). However, as with self-reporting AA men, these geographic differences attenuated when comparing mean YRI estimates in non-ethnic EA research subjects, *p* = 0.06.

**Table 1:** Self-reported race and mean individual ancestry estimates by races and geographic region.

Self Reported Race	Geographic Region	Mean YRI (African) ancestry (%)	Mean CEU (European) ancestry (%)	Mean ASI (Asian) ancestry (%)	p-value**
**AA** **n = 1043**	Louisiana n = 594	86.9	11.9	1.2	0.03
	North Carolina n = 449	89.5	9.3	1.2	
**EA** **n = 1063**	Louisiana n = 582	1.8	96.9	1.3	0.001
	North Carolina n = 481	0.8	98.4	0.8	
**Non-ethnic AA** * **n = 930**	Louisiana n = 485	89.2	9.5	1.3	0.78
	North Carolina n = 445	89.4	9.4	1.2	
**Non-ethnic EA** * **n = 824**	Louisiana n = 354	1.5	97.3	1.2	0.002
	North Carolina n = 470	0.8	98.6	0.6	

*includes *ONLY* individuals reporting “no” ethnicity membership.

**One-way multivariate analysis of variance (MANOVA) models comparing mean CEU and YRI ancestry estimates between research subjects in North Carolina and Louisiana.

### Self-Reported Race, Ethnicity and Individual Ancestry

Mean IA estimates by race and ethnicity are shown in Table 2. MANOVA models showed mean ancestry estimates for YRI and CEU in self-reporting AA identifying as Cajun only, Creole only or both Cajun and Creole significantly differed from those men identifying as non-ethnic AA research subjects, *p*<0.00001, *p*<0.00001, *p* = 0.03, respectively. Mean CEU and YRI ancestry differences in self-reported EA were seen when comparing Hispanics only to non-ethnic EA research subjects (p<0.0001).

**Table 2:** Mean individual ancestry estimates by ethnicity for Louisiana only*.

Self-Reported Race	Self Reported Ethnicity, Louisiana only (n)	Mean YRI (African) ancestry (%)	Mean CEU (European) ancestry (%)	Mean ASI (Asian) ancestry (%)	p-value**
**AA**	Cajun (10)	45.2	53.0	1.8	<0.00001
	Creole (71)	77.8	21.1	1.1	<0.00001
	Cajun and Creole (7)	70.7	29	0.4	0.02
	No ethnicity reported (485)	89.2	9.5	1.3	referent group
**EA**	Cajun (187)	1.4	97.7	0.9	0.68
	Cajun and Creole (6)	0.7	98.9	0.5	0.86
	Hispanic/Latino (15)	14.8	77.8	7.3	<0.00001
	No ethnicity reported (354)	1.5	97.2	1.2	referent group

*all estimates and *p*-values include only Louisiana individuals who answered either “yes” or “no” to *ALL* ethnicity questions; individuals reporting “don't know” or missing ethnicity information were not included. Research participants reporting two or more ethnicities (aside from Cajun and Creole) were not included in estimates or statistical tests due to small sample size (n≤2).

**one way MANOVA.

### Principal Components Analysis

**Figure 1:**
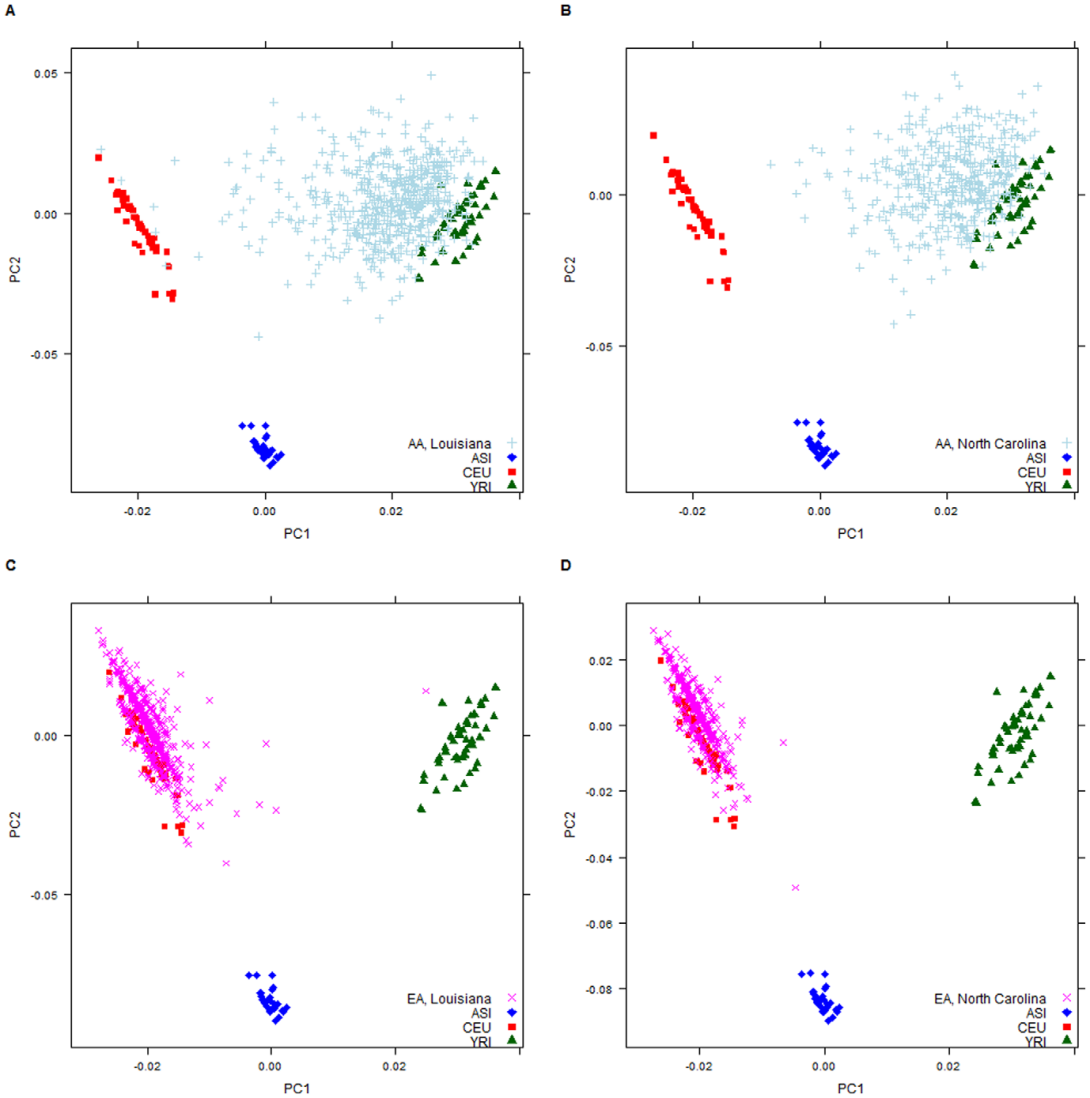
Clustering of self-reporting AA and EA research subjects and HapMap CEU, YRI, ASI (JPT+CHB) populations based on PC1 and PC2. Plots of AA and EA research participants in Louisiana (**a,c**) and North Carolina (**b,d**).

PCs 1–4 sequentially (cumulatively) accounted for 73.2, 6.6 (79.8), 2.8 (82.6) and 2.7 (85.3) percent of the total genetic variation. The amount of variation explained after PC2 appears minimal with constant scree plots (a line segment plot that shows the fraction of total variance in the data as explained by each PC) of the eigenvalues becoming essentially constant at PC3 onward [30]. Scatter plots of PC1 and PC2 from PCA segmented by race and location revealed that AA show a wider range of European and Asian ancestry than European Americans in both Louisiana (Figure 1a) and North Carolina (Figure 1b) with AA from Louisiana showing the most dispersion. Self-reporting EA research subjects form distinctive clusters in Louisiana and North Carolina (Figures 1c and 1d, respectively) and on average the genetic composition for these groups is most similar to their HapMap counterparts. PC distribution by state was similar for PC1 but differed for PC2 (p<0.02).

Due to the self-reported and genetic diversity in Louisiana, the Wilcoxon rank sum was used to assess differences in the top two PCs by ethnicity. PC1 and PC2 differed significantly (p<0.001 and p<0.028, respectively) between Creole and non-ethnic AA. As with IA estimates other significant differences in CEU were observed in a series of exploratory analyses. For example, when comparing subjects who reported both Cajun and Creole to the non-ethnic AA, PC1 (p<0.05) and PC2 (p<0.015) were significantly different as was PC1 when comparing Cajun alone and non-ethnic African American men, p<0.0001. PCs 1 and 2 were differentially distributed (p<0.0001 for both PCs) between Hispanic and non-ethnic EA.

**Figure 2:**
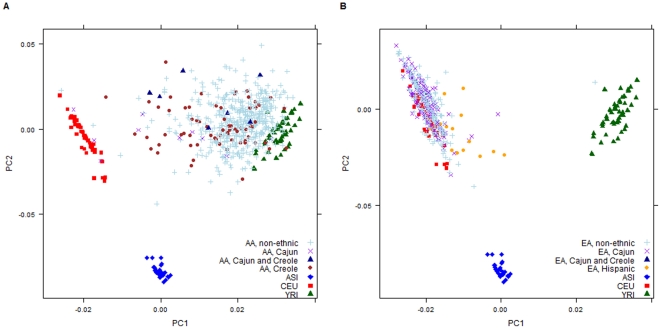
Clustering of self-reporting AA and EA research subjects in Louisiana with Hapmap, CEU, YRI and ASI (JPT+CHB) populations based on PC1 and PC2. Plots of AA (**a**) and EA (**b**) with self-reported ethnicity.

The ethnicity specific PCs for Louisiana AA and EA are shown in Figures 2a and 2b, respectively. For AA research subjects PC1 and PC2 separate the reported ethnicities with reasonable clarity and individuals showing varying degrees of admixture from most (Cajun and Creole) to least (non-ethnic AA) are visible. In contrast, EA research participants from Louisiana show minimal distance from one another and cluster closely with their Hapmap CEU counterpart.

## Discussion

Ancestry estimates, derived using MCMC clustering algorithm implemented in STRUCTURE, were used to evaluate genetic composition between and within self-reported races in North Carolina and Louisiana. Mean IA differences by race between states were small (<3%) but statistically significant. Given the large mean IA differences across ethnic groups within Louisiana genetic heterogeneity appeared to be the main contributing factor to the mean IA differences by state. However, it is possible that other factors are driving these differences. For example, a similar effect would be seen if research participants self-reporting as AA derive ancestry from different areas in Africa and the chosen reference population, YRI, is more similar to that found in one state versus the other.

The limitations in using self-reported race to reveal population genetic substructure have been shown repeatedly [14], [17]. Results from these studies demonstrate a reduced probability of finding genetic association in epidemiologic studies if population stratification is not measured adequately [14], [15]. While the magnitude of the effect of population structure on case-control studies has been debated, larger bias can be introduced when individuals of the same race/ethnicity but from different geographic areas are combined [15], [17], [31]. Thus, both genetic ancestry and self-reported race and ethnicity must be characterized in cohort and case-control studies. Ancestral proportions are dependent on reference populations used in estimation, the AIMs selected and the method of estimation, therefore the limitations of our study are those common to any study involving the estimation of genetic ancestry. Additional populations other than the ones used in this study (African, European and Asian) may be warranted, however most research has shown that only two populations are representative of the ancestral populations of European and African American individuals from the United States.

Characterization of the genetic background that exists at both the population and individual level offers the promise of an improved understanding of the underlying factors leading to differential disease susceptibility and differential response to pharmacological agents, and to disentanglement of the complex interaction between genetic and environmental factors in the disease phenotype. The topics of race and ethnicity continue to be of considerable interest and debate with respect to scientific and medical research [30], [31], [32]. We have found that genetic ancestry varies significantly by and within geographic region among individuals self-identifying as belonging to the same racial group. The well-characterized genetic background of the PCaP cohort will now allow examination of the association of self-reported race, ethnicity and genetic ancestry with CaP aggressiveness when considering socioeconomic, genetic and environmental factors with the ultimate goal of more fully understanding CaP racial disparities.

## Supporting Information

Table S1 **Allele frequencies for AIMs in PCaP cohort by self-reported race.**
(DOC)
