# Supplementary material for: Genetic Ancestry, Self-Reported Race and Ethnicity in African Americans and European Americans in the PCaP Cohort
Source: PLoS One. 2012 Mar 27;7(3):e30950. doi: 10.1371/journal.pone.0030950 (PMC3313995; doi:10.1371/journal.pone.0030950)
Supplement: Table S1 — Allele frequencies for AIMs in PCaP cohort by self-reported race. (DOC) [file pone.0030950.s001.doc]

| **rs id** | **Chr** | **BP Position** | **Reference allele** | **Allele frequency, Self-Reported AA (%)** | **Allele frequency,Self-Reported EA (%)** | **Allele frequency δ EA/AA**  **(%)** |
| --- | --- | --- | --- | --- | --- | --- |
| **rs6672228** | 1 | 25430739 | T | 57.7 | 0.70 | 57.2 |
| **rs1028304** | 1 | 175325703 | C | 89.9 | 51.0 | 38.9 |
| **rs342780** | 1 | 227385141 | A | 54.9 | 0.50 | 54.4 |
| **rs962646** | 2 | 12304711 | G | 58.1 | 1.40 | 56.8 |
| **rs2373860** | 2 | 40489535 | T | 89.2 | 49.7 | 39.4 |
| **rs6750678** | 2 | 56707694 | C | 51.4 | 99.3 | 47.9 |
| **rs4675545** | 2 | 206392099 | A | 50.7 | 99.1 | 48.4 |
| **rs7622739** | 3 | 124832181 | G | 88.0 | 40.6 | 47.4 |
| **rs833282** | 3 | 183071456 | C | 87.9 | 43.0 | 44.8 |
| **rs6827593** | 4 | 52642464 | T | 51.8 | 01.0 | 50.9 |
| **rs10016143** | 4 | 149835011 | G | 61.4 | 00.9 | 60.4 |
| **rs16891982** | 5 | 33987450 | C | 81.2 | 05.3 | 75.9 |
| **rs6920128** | 6 | 3157381 | G | 59.7 | 00.7 | 59.0 |
| **rs1341567** | 6 | 76684896 | C | 58.3 | 01.2 | 57.1 |
| **rs1321271** | 6 | 132513304 | G | 53.8 | 99.2 | 45.3 |
| **rs200153** | 6 | 143479138 | G | 88.7 | 51.7 | 36.9 |
| **rs7802165** | 7 | 3482441 | C | 77.3 | 85.7 | 8.3 |
| **rs2188405** | 7 | 92744573 | T | 90.1 | 55.1 | 35.0 |
| **rs4308654** | 7 | 113258417 | A | 88.8 | 46.5 | 42.4 |
| **rs6962773** | 7 | 146145059 | A | 62.2 | 01.2 | 60.8 |
| **rs10125239** | 9 | 98323553 | C | 52.4 | 01.0 | 51.5 |
| **rs10995842** | 10 | 65643820 | G | 89.4 | 45.5 | 43.9 |
| **rs670283** | 10 | 105917100 | G | 59.2 | 01.2 | 58.1 |
| **rs10506122** | 12 | 36985499 | T | 88.2 | 58.4 | 29.7 |
| **rs17662023** | 12 | 51220616 | T | 90.7 | 48.0 | 42.6 |
| **rs17835503** | 12 | 77371908 | T | 88.3 | 45.8 | 42.5 |
| **rs7980055** | 12 | 107717470 | G | 84.9 | 46.0 | 39.1 |
| **rs4534620** | 12 | 121975121 | T | 52.5 | 01.3 | 51.1 |
| **rs8000445** | 13 | 66272179 | C | 51.2 | 0.9 | 50.2 |
| **rs11618812** | 13 | 92847878 | A | 91.7 | 52.0 | 39.7 |
| **rs12855009** | 13 | 105661264 | C | 91.4 | 53.8 | 37.6 |
| **rs10483929** | 14 | 79193506 | C | 56.9 | 99.1 | 42.2 |
| **rs10520735** | 15 | 91874783 | G | 86.7 | 24.3 | 62.3 |
| **rs16951604** | 16 | 51405969 | T | 54.4 | 0.7 | 53.6 |
| **rs9893667** | 17 | 55876335 | C | 65.5 | 1.1 | 64.5 |
| **rs1941267** | 18 | 28683311 | G | 55.3 | 1.4 | 54.0 |
| **rs7246566** | 19 | 38281458 | T | 85.8 | 36.1 | 49.7 |
| **rs838145** | 19 | 53940542 | A | 91.0 | 58.1 | 33.0 |
| **rs1407020** | 20 | 31296129 | G | 54.7 | 0.9 | 53.8 |
| **rs6070851** | 20 | 57584062 | T | 88.2 | 41.5 | 46.7 |
